# Master transcriptional regulator SaeS in *Staphylococcus aureus* contributes to staphyloxanthin biosynthesis to promote survival during invasive infection

**DOI:** 10.1080/21505594.2025.2580159

**Published:** 2025-10-31

**Authors:** Eunhwan Bae, Donggyu Kim, Minjin Kim, Anna Kang, Jinwook Shin, Younghoon Kim, Minhye Shin

**Affiliations:** a Department of Microbiology, College of Medicine, Inha University, Incheon, Republic of Korea; b Program in Biomedical Science and Engineering, Inha University, Incheon, Republic of Korea; c Department of Agricultural Biotechnology, Research Institute of Agriculture and Life Science, Seoul National University, Seoul, Republic of Korea

**Keywords:** *Staphylococcus aureus*, SaeRS, staphyloxanthin, zinc

## Abstract

*Staphylococcus aureus* is a major human opportunistic pathogen that causes significant morbidity and mortality, particularly in immunocompromised individuals. SaeRS is a two-component system in *S. aureus* that regulates signal transduction related to virulence, including hemolysis and coagulation. Metal ions are essential nutrients that support bacterial virulence and survival against host immune cells and are intricately interconnected with regulatory systems. The SaeRS system has long been studied for its function in bacterial virulence and invasive infections. However, its interactions with other regulators and metal ions remain unelucidated. Thus, this study evaluated the effects of the *S. aureus* SaeRS system on virulence, specifically its association with oxidative stress resistance and staphyloxanthin (STX) production. *saeS* deletion reduced STX production via the SigB-CrtOPQMN pathway, increasing vulnerability to oxidative stress and susceptibility to host immune cells. Supplementation with metal ions, specifically zinc, inhibited STX-associated gene expression, attenuating antioxidative activity *in vitro*. Experiments on mice with *S. aureus* bloodstream infection verified that SaeS was crucial for bacterial survival *in vivo*. Furthermore, zinc contributed to weakened bacterial virulence and altered host immune defense mechanisms. Collectively, our results established a novel mechanistic interconnection between SaeRS and STX biosynthesis and demonstrated that SaeRS inhibition combined with zinc supplementation promotes innate immune system-mediated killing of *S. aureus*.

## Introduction

*Staphylococcus aureus* is an opportunistic pathogen capable of causing a wide spectrum of diseases, ranging from superficial skin infections to life-threatening invasive conditions [1]. Notably, it is among the leading causes of bloodstream infections, with high morbidity and mortality rates in developed countries [2]. Despite several studies investigating *S. aureus* pathogenesis, preventive measures, including vaccines, remain limited. Furthermore, widespread drug resistance to multiple antibiotics hinders the effective management of these infections [3].

The virulence of *S. aureus* is driven by multiple factors, including multilayered biofilm formation, secretion of pore-forming toxins like α-hemolysin and coagulases, and innate immune evasion strategies such as the production of staphyloxanthin (STX) [4–6]. These factors facilitate bacterial invasion of host cells and modulate the host’s immune response. The expression of virulence factors is highly dependent on transcriptional regulators, which are also affected by environmental cues, including the availability of specific nutrients, particularly dietary metal ions [7,8].

To control virulence in their host, *S. aureus* has evolved an interconnected regulatory network of two-component systems (TCS) essential for survival and virulence modulation under certain environmental conditions [9]. One such TCS is SaeRS, a master transcriptional regulator for over 20 regulons, including hemolysins, coagulase, and surface proteins [10]. It is encoded within the *saePQRS* operon transcribed from P1 and P3 promoters and comprises the sensor histidine kinase SaeS, response regulator SaeR, and auxiliary genes encoding SaeP and SaeQ. Although SaeS has been recognized for its function against host innate immune responses and its role in invasive infections, cognate Sae signals and regulatory mechanisms remain unelucidated [11].

The SaeRS TCS is affected by many molecules, including human neutrophil peptides, calprotectin, lipoteichoic acid, and metal ions [10]. Metal ions derived from the host’s diet can influence pathogen survival during infection, depending on their availability in host tissues [12]. Alterations in metal homeostasis, particularly decreased levels of dietary Zn, were reported to increase infection risk [13]. Furthermore, an *in vitro* assay demonstrated that Cu, Zn, and Fe inhibited SaeS autophosphorylation [14]. However, the effect of metal ions on *S. aureus* infection *in vivo* and the SaeRS TCS remains unknown

In this study, we discovered that *saeS* deletion attenuated STX biosynthesis by decreasing the expression of *crt* and *rsb* operon genes. We also investigated the effect of metal ions on *S. aureus* pathogenicity and the relationship between Zn and STX expression. Experiments using an *S. aureus* bloodstream infection mouse model revealed that SaeS was crucial for bacterial survival and that Zn supplementation weakened bacterial virulence and altered host immune defense mechanisms.

## Materials and methods

### Ethical approval statement

The use of human-derived materials (whole blood) in this study was reviewed and approved by the Institutional Review Board (IRB) of Inha University (IRB Approval No. 230,527-1AC). All procedures were conducted in accordance with the Bioethics and Safety Act and the Personal Information Protection Act of the Republic of Korea. All experimental protocols were in accordance with the Declaration of Helsinki (2022). Prior to enrollment, all participants were provided with the information concerning the purpose, study method, expected and unexpected side effects, and voluntarily provided written consent. Whole blood samples were obtained from healthy volunteers with written informed consent, and all samples were anonymized prior to use.

All animal procedures were conducted in accordance with the relevant guidelines and regulations of the Republic of Korea. A total of 30 mice were used in this study, and all efforts were made to minimize animal suffering and to use the minimum number of animals required to achieve statistical significance. The experimental protocol was reviewed and approved by the Institutional Animal Care and Use Committee (IACUC) of the Lee Gil-Yeo Cancer and Diabetes Research Center, Gachon University, Incheon, South Korea (Approval Number: LCDI-2022–0060). All animal experiments were performed in certified facilities under the supervision of qualified personnel and conducted in accordance with the ARRIVE (Animal Research: Reporting of In Vivo Experiments) guidelines.

### Bacterial strains and growth conditions

Supplementary Table S1 details the bacterial strains used in this study. *S. aureus* was cultured at 37° in brain heart infusion (BHI) broth (CM1135, Oxoid; Thermo Scientific, Waltham, MA, USA). *Escherichia coli* was cultured at 37° in Luria – Bertani (LB) broth (#244620, BD, Franklin Lakes, NJ, USA). *S. aureus* strain Newman-*ΔsaeS* was cultured at 37° in BHI broth supplemented with 200 μg/ml erythromycin, and *S. aureus* strain Newman-*ΔsaeS* complement was cultured in BHI broth supplemented with 200 μg/ml erythromycin and 10 μg/ml chloramphenicol. When incubating on RPMI 1640 (#SH30027.01; HyClone, UT, USA), strains cultured overnight in BHI broth were washed twice with RPMI 1640 medium, diluted to an optical density at 600 nm (OD_600_) of 0.05, and incubated in fresh RPMI 1640 medium.

### Genome editing using the CRISPR-Cas9 system

To construct pCasSA-*∆saeS* plasmid for *saeS* deletion in *S. aureus* Newman, the CRISPR/Cas9 system was applied using pCasSA (#98211; Addgene, Watertown, MA, USA) as the backbone, as described in Chen et al. [15]. The expression of a single synthetic guide RNA was driven by the *cap 1A* promoter. The CRISPR RNA (crRNA) sequence was predicted using CRISPOR (http://crispor.tefor.net/) and inserted together with the trans-activating RNA (tracrRNA) [16]. Deletion of the *saeS* gene was achieved through homologous recombination-mediated repair following a double-strand DNA break, facilitated by expression of the repair arms. The constructed pCasSA-*∆saeS* plasmid was electroporated into *S. aureus* RN4220, which possesses a defective restriction-modification system, at 2.1 kV/cm, 25 µF, and 200 ohms. The final plasmid was then extracted from *S. aureus* RN4220 and transformed into *S. aureus* Newman. Genome editing was verified by polymerase chain reaction (PCR) and sequencing (Supplementary Figure S1).

*saeS* was overexpressed in *S. aureus* Newman*-∆saeS* using the pCasSA plasmid. To generate the SaeS complement strain, the *cas9* locus inserted under the *rpsL* promoter was replaced with *saeS* (Supplementary Figure. S2). The plasmids and primers used in this study are detailed in Supplementary Tables S1 and S2, respectively.

### Structural modeling and docking analysis

The complete SaeS sequences of the WT, mutant 1, and mutant 2, consisting of 351, 318, and 210 amino acids respectively, were obtained from whole genome sequencing results. The protein homology models of SaeS WT was constructed using AlphaFold Protein Structure Database (https://alphafold.ebi.ac.uk/) [17] and its mutants were constructed using SIWSS-MODEL (https://swissmodel.expasy.org/) [18] based on the constructed SaeS WT protein structure as a template. It is noted that the amino acid sequences of the mutant SaeS protein included premature stop codons due to nucleotide mutations and frame shift. Therefore, protein structure prediction for the mutants was conducted using the truncated sequences up to the stop codon. These sequences were input into SWISS-MODEL, which aligned the target sequence with the template structure, and generated a 3D model of the target protein. The resulting homology models were visualized using UCSF ChimeraX version 1.10.1 (https://www.rbvi.ucsf.edu/chimerax/).

The tertiary structure of the nucleic acid sequence was predicted using the RNAComposer web server [19]. The 3D structure of the SaeR protein (PDB ID: 4QWQ) was retrieved from the Protein Data Bank (PDB), which corresponds to the DNA-binding domain of the response regulator SaeR from *Staphylococcus aureus*. The SBS (SaeR binding site) upstream of the *hla* gene was based on a previously reported sequence by Liu, Qian et al. [10]. In contrast, the putative SBS of *rsb* was predicted using the sequence pattern described by Liu, Qian et al. [10], suggesting a potential interaction between SaeR and the *rsb* promoter region. All protein – nucleic acid interactions were predicted using the HDOCK server [20]. Predicted complexes were visualized using the Discovery Studio Visualizer software. The SaeR–*saeP* binding model, which includes a known SBS site, was used as a control.

### Catalase activity assay

Catalase activity assays were performed based on a modified version of the colorimetric method developed by Hadwan et al. [21], to evaluate oxidative stress resistance in *S. aureus* strains. *S. aureus* grown overnight in BHI broth was washed twice with RPMI1640 medium. Fifty microliters of the washed culture were added to 5 ml of the same medium and incubated overnight at 37°C with shaking. After incubation, bacterial cells were harvested by centrifugation at 4,000 × g for 15 min, washed three times with phosphate-buffered saline (PBS), and adjusted to an OD₆₀₀ of 1.0. Cell lysis was then performed by bead-beating (vortexing for 1 min and ice for 1 min, repeated three times), and the resulting lysates were used as catalase assay samples. Ferrous ammonium sulfate (FAS) and sulfosalicylic acid (SSA) were prepared at 10 mM concentration by dissolving them in 7% (v/v) glacial acetic acid. Equal volumes of FAS and SSA solutions were mixed at a 1:1 ratio immediately before use to prepare the working reagent. In a 96-well microplate, 100 µL of 5 mM hydrogen peroxide solution was mixed with 20 µL of the prepared bacterial samples in each well. The plate was incubated at 37°C for 5 min. Then, 130 µL of the freshly prepared working reagent was added and mixed thoroughly. The plate was further incubated at 25°C for 5 min, and the absorbance was measured at 490 nm using a microplate reader.

### Coagulation assay

Staphylococcal virulence assays for coagulation, hemolysis, and STX production were performed per Shin et al. [22] with slight modifications. For the coagulation assay, five colonies of overnight-grown *S. aureus* Newman on BHI agar were picked using a sterilized loop and mixed with 500 μl of lyophilic rabbit plasma (#MB-88030; KisanBio, Seoul, South Korea) in a 1.5-ml microcentrifuge tube. The plasma mixture was incubated at 37° for 24 h, and coagulation was assessed by gently tapping the tube.

### Hemolysis assay

For the hemolysis assay, *S. aureus* grown overnight in BHI broth was washed twice with RPMI 1640 medium. Fifty microliters of the washed culture were added to 5 ml of the same medium and incubated overnight at 37° with shaking. The overnight culture (1 ml) was centrifuged at 13,000 rpm at room temperature for 5 min, and 10 µl of the resulting supernatant was added to 1 ml of 2% (v/v) defibrinated sheep blood (#MB-S1876; KisanBio, Seoul, South Korea) in 0.9% (w/v) NaCl. The blood mixture was incubated at 37° for 15 min. After incubation, the tubes were centrifuged at 3,500 rpm for 5 min, and the supernatant was diluted 1:10 with 0.9% (w/v) NaCl before being transferred to a 96-well plate. Absorbance was measured at 405 nm using an Epoch Microplate Spectrophotometer (BioTek Instruments, Winooski, VT, USA).

### STX production assay

For the STX production assay, *S. aureus* grown overnight in BHI broth was washed twice with RPMI 1640 medium. Fifty microliters of the washed culture were added to 5 ml of the same medium and incubated overnight at 37° with shaking. The overnight culture was centrifuged at 13,000 rpm for 1 min, washed twice with 0.9% (w/v) NaCl, and the resulting cell pellets were collected. Cold ethanol extraction method was applied to extract STX by adding 200 µl of ice-cold absolute ethanol, incubating for 30 min, and centrifuging at 13,000 rpm for 5 min. The resulting supernatant was transferred to a well of a 96-well plate, and absorbance was measured at 450 nm using a microplate reader.

### Quantitative reverse transcription PCR (qRT-PCR)

Isolation of RNA was performed using a RNeasy Mini Kit (#74104, Qiagen, Hilden, Germany) following the manufacturer’s protocol with slight modifications. *S. aureus* was grown overnight in RPMI 1640 medium at 37°C with shaking. After centrifugation, the pellet resuspended in RLT buffer, and ice-cold glass beads were added. The mixture was vortexed for 1 min and then placed on ice for 1 min. This process was repeated three times to ensure complete cell disruption. RNA concentration was measured using a NanoDrop One Microvolume UV-Vis Spectrophotometer (#13–400-518; Thermo Scientific). The RNA concentration ranged from 50 to 150 ng/µL, extracted from 1 mL of bacterial pellet. The integrity of the extracted RNA was evaluated by agarose gel electrophoresis prior to cDNA synthesis. cDNA was synthesized from the purified RNA using an iScript cDNA Synthesis Kit per the manufacturer’s protocol (#1708890, Bio-Rad, Hercules, CA, USA).

qRT-PCR assay was performed mixing SsoAdvanced Universal SYBR Green Supermix (#1725270; Bio-Rad) with 125 ng/μl of cDNA and 10 μM of each primer in a final reaction volume of 10 µl. Quantitative PCR (Bio-Rad, CFX96 Real-Time PCR Detection System) was performed using the housekeeping gene *gyrB*. The primer sequences are presented in Supplementary Table S2.

### H_2_O_2_ resistance assay

*S. aureus* was cultured overnight in BHI broth, washed twice with RPMI 1640 medium, diluted to an initial OD_600_ of 0.05, and cultured in 5 ml of fresh RPMI 1640 medium at 37°C until the OD_600_ reached 1.0–1.3 (approximately 7–8 h). Then, 5 µl of 30% H_2_O_2_ was added to 100 μl of the culture (final concentration: 1.5% [v/v]), and the mixture was incubated at 37°C for 1 h with shaking. Finally, the reaction mixture was serially diluted with 0.9% NaCl and spread on BHI agar to determine the number of surviving colony-forming units (CFUs).

### Whole blood killing assay

*S. aureus* was cultured overnight in BHI broth, washed twice with RPMI 1640 medium, diluted to an initial OD_600_ of 0.05, and cultured in 5 ml of fresh RPMI 1640 overnight at 37° in 14-ml round tubes with shaking. After washing the cells twice with phosphate-buffered saline (PBS), 50 μl of human whole blood collected in heparinized tubes was mixed with 50 μl of *S. aureus* (10^4^ CFU/ml) and incubated at 37°C for 4 h with shaking. The reaction mixture was serially diluted with 0.9% NaCl and spread onto BHI agar to determine the number of surviving CFUs.

### Plasma killing assay

The whole blood was collected from mice using heparinized tubes to prevent coagulation and was kept at 4°C until analysis. To isolate plasma, the anticoagulated blood was centrifuged at 2,000 × g for 15 minutes at 4°C. Plasma was collected and used in place of whole blood in the whole blood killing assay protocol. Specifically, 50 μl of isolated plasma was mixed with 50 μl of *S. aureus* (10^4^ CFU/ml), and the mixture was incubated at 37°C for 4 h with shaking. The reaction mixture was serially diluted with 0.9% NaCl and spread onto BHI agar to determine the number of surviving CFUs.

### Animal study

Female C57BL/6 mice (5 weeks old, 15–17 g) were purchased from DBL (Chungbuk, South Korea). Mice were housed in groups of five per cage under a 12-hour light/dark cycle at 24°C and acclimated for one cycle at the Animal Care Center. A priori power analysis using G*Power 3.1.9.7 (effect size f = 0.8, a = 0.05, power = 0.8) [23] indicated that 26 animals per group would be required. However, due to ethical considerations and prior in vitro findings demonstrating clear group differences, the number of animals was reduced to five per group.

Starting from the age of 6 weeks, the mice were initially divided into three groups, including mock infection, *S. aureus*-WT infection, and *S. aureus-∆saeS* infection, and further divided into two groups with untreated water and water supplemented with Zn. For Zn supplementation, the mice were allowed to drink water supplemented with 250 ppm ZnSO_4_ *ad libitum* for approximately 6 weeks [24]. At 8 weeks of age, the bloodstream infection was induced by inoculation with 0.1 ml of *S. aureus* culture (10^7^ CFU) via a vein at the tip of the tail. Their survival and body weight were monitored for approximately 22 days. Six cages (corresponding to each group) and five mice were numbered, and bacterial inoculation and body weight measurements were conducted on randomly selected individuals. No animals were excluded from study. Outcome assessment and data analysis were performed by investigators blinded to group allocation. Group allocation and the order of treatment were randomized to minimize selection bias and confounding factors. At the experimental endpoint on day 22, the mice were euthanized in the order of their assigned numbers, and organs, tissues, and blood were surgically collected. The harvested organs were divided into two parts: one used immediately for infection rate assessment and the other immediately frozen in liquid nitrogen and stored at −80°C for subsequent Gram and hematoxylin/eosin stainings. The collected blood was processed to separate the serum, which was also immediately stored at −80°C.

To determine the infection rate, organ tissue was transferred into 1.5-ml tubes and homogenized. The cell homogenate was serially diluted with PBS, and 100 μl was spread onto a BHI agar plate for overnight incubation at 37°. The number of CFUs was counted the following day. Samples of the frozen organs were placed in Tissue-Tek OCT Compound (4583; Sakura Finetek, Torrance, CA, USA) and sliced into 7-μm thick sections using a Shandon Cryotome FE cryostat (Thermo Scientific). Following Gram and hematoxylin/eosin stainings, the sections were analyzed using a light microscope (BX53, Olympus, Tokyo, Japan).

All animals were monitored daily for general health and any signs of distress. Humane endpoints included weight loss, a rough hair coat, reduced activity, and abnormal posture. Animals meeting these criteria were humanely euthanized using CO_2_ inhalation followed by cervical dislocation.

### Analysis of inflammatory cytokines

Blood from the sacrificed mice was allowed to clot for 30 min at room temperature. Then the serum was collected following centrifugation at 3,500 rpm for 10 min at 4°C and stored at −80°C for analysis of the levels (pg/ml) of interleukin (IL)-10, IL-6, and tumor necrosis factor alpha (TNF-α) using a Mouse Premixed Multi-Analyte kit (#LXSAMSM-05, R&D Systems, Bio-Techne, Minneapolis, MN, USA) on a Luminex instrument (LABISKOMA, Seoul, South Korea).

### Statistical analysis

Experimental data were evaluated using GraphPad Prism v9 software (GraphPad Software, San Diego, CA, USA) with unpaired Student *t*-test and one-way ANOVA. Survival analyses were performed using Kaplan – Meier survival curves (log-rank test). Statistical significance was assigned as p-values: **** < 0.0001; *** < 0.001; ** < 0.01, * < 0.05. Most *in vitro* and *in vivo* experiments were performed at least three biological replicates, as noted in the figure legends.

## Results

### SaeS deletion reduces virulence gene expression in *S.* *aureus*

**Figure 1.**
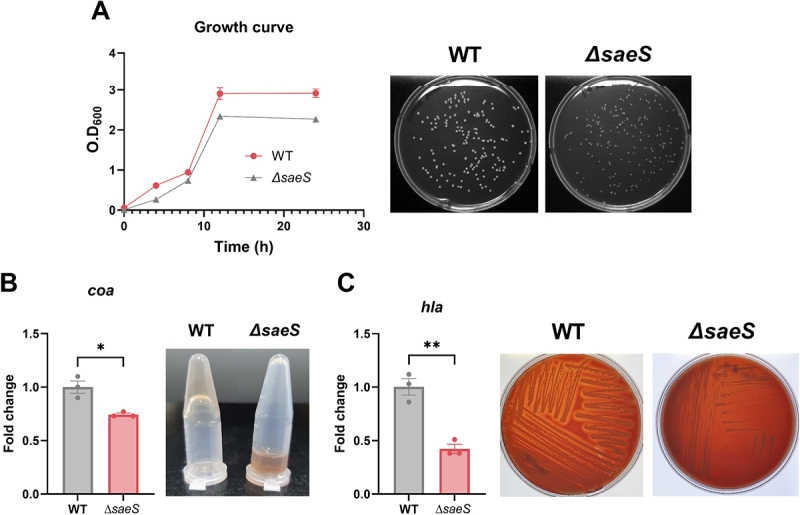
*SaeS* deletion suppresses virulence expression in *S. aureus*. (A) Growth and colony formation of *S. aureus* wild-type (WT) and *Δsaes* mutant strains. (B and C) Coagulation (B) and hemolysis (C) resulting from *coa* and *hla* expression, respectively. Significant differences between groups were assessed using Student *t*-test and are indicated by asterisks (P-values: *  < 0.05, and ** < 0.01). Data are presented as mean ± SD of biological replicates from three independent experiments.

Previously, we obtained two colonies exhibiting growth defects on blood agar medium [25,26], presenting as pinpoint colonies with impared STX biosynthesis, antioxidant defense, and hemolysis (Supplementary Figure S3(A,B)). Whole genome sequencing followed by single nucleotide polymorphism (SNP) analysis revealed that both clones harbored a mutation in the *saeS* gene (Supplementary Figure S3(C,D)). The SaeRS TCS in *S. aureus* is a well-known transcriptional regulator associated with various virulence factors, including α-hemolysin (encoded by *hla*), coagulases (encoded by *coa*), and surface proteins [10]. Notably, several studies have demonstrated that the deletion of the entire SaeRS system reduced coagulation and the expression of toxic shock syndrome toxin 1 and α-hemolysin, but only a few studies have investigated SaeS-specific null mutants [27–29]. To determine the contribution of SaeS to altered bacterial pathogenesis, we generated a SaeS-null mutant (*ΔsaeS*) and evaluated its pathogenic phenotypes compared with the WT (Figure 1). The colonies of the *ΔsaeS* strain were smaller than those of the WT strain on solid agar. Furthermore, the bacterial cell density was slightly decreased in the stationary phase in liquid medium culture (Figure 1(A)). Additionally, *ΔsaeS* exhibited attenuated coagulation and hemolysis with reduced *coa* and *hla* expression (Figure 1(B,C)), supporting previous findings [30]. These data suggested that staphylococcal SaeS would be associated with bacterial fitness and virulence.

### SaeS deletion reduces STX synthesis associated with oxidative stress resistance

**Figure 2.**
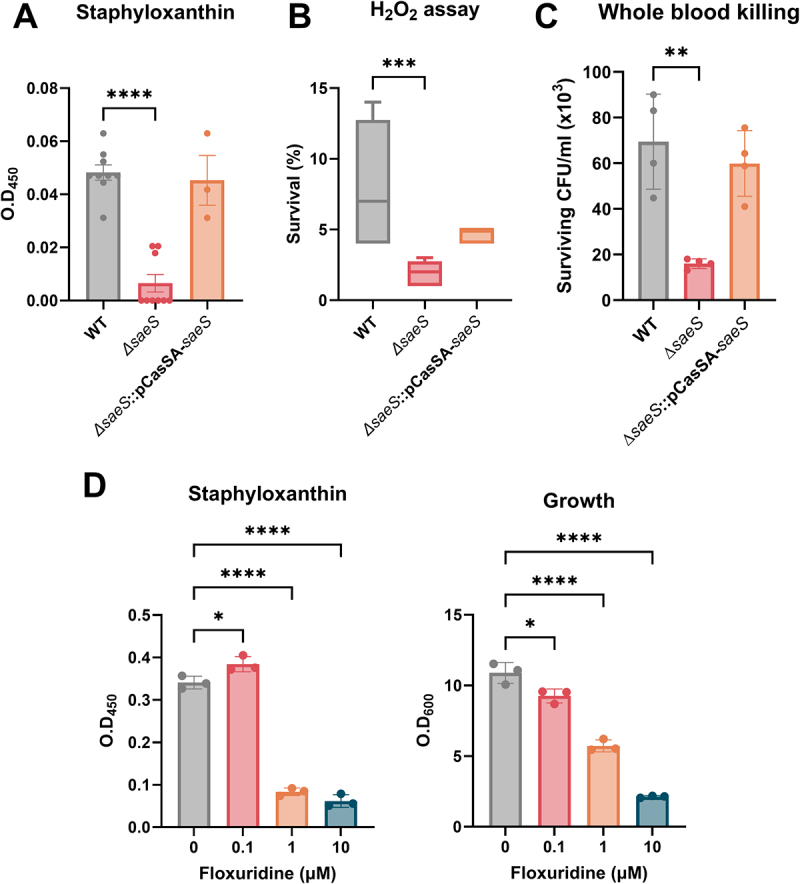
*SaeS* deletion suppresses STX synthesis in *S. aureus* Newman. (A) STX production in *S. aureus* Newman (WT) and *ΔsaeS*, *Δsaes*-complemented strain. Bacterial resistance to oxidative stress based on survival in H_2_O_2_ (B) and whole blood killing (C). (D) Inhibition of STX production by floxuridine as a SaeS inhibitor. One-way anova with Dunnett post hoc analysis was used to assess significant differences among the groups (P-values: *  < 0.05** < 0.01, *** < 0.001, and **** < 0.0001). Data are presented as mean ± SD of biological replicates from three to nine independent experiments.

STX is a yellow-to-orange carotenoid pigment that uses its antioxidative activity to play a major role in protecting bacteria against neutrophil killing [31]. Besides hemolysis and coagulation, *ΔsaeS* showed distinguishable defects in pigment production (Figure 2(A)). To confirm its antioxidative activity, we analyzed bacterial resistance against H_2_O_2_. *∆saeS* was killed by the superoxide radical, showing that STX production mediated by SaeS conferred antioxidative survival advantages in *S. aureus* (Figure 2(B)). Additionally, *saeS* deletion resulted in a reduced number of surviving bacteria when treated with whole blood, demonstrating the involvement of SaeS in bacterial survival against phagocytic killing (Figure 2(C)). It is noted that no significant effect of *saeS* deletion was observed using blood plasma, ruling out the involvement of complement proteins present in plasma (Supplementary Figure S4). To further determine whether the decreased oxidative stress resistance in the *ΔsaeS* mutant results from other factors such as catalase beyond the reduction in STX production, we performed a catalase activity assay using WT, *∆saeS*, and complemented strains (*∆saeS*::pCasSA-*saeS*). No significant differences in catalase activity were observed among the strains, excluding a potential association between SaeS and catalase in *S. aureus* (Supplementary Figure S5). In addition, analysis of the complemented strain showed that restoration of the *saeS* gene restored the WT phenotype (Figure 2(A–C)). These findings support the role of *saeS* in STX synthesis, which contributes to oxidative stress resistance. To verify the reduced STX production resulting from SaeS inhibition, floxuridine, a known inhibitor of the Sae system [32], was added to the bacterial culture. The inhibition of STX production was more pronounced, particularly at concentrations of 1 and 10 μM, compared to its effect on bacterial growth (Figure 2(D)). Collectively, these results suggest that SaeS is involved in STX biosynthesis regulation, which is associated with oxidative stress resistance.

### SaeS deletion suppressed *crt* and *rsb* operon expression

**Figure 3.**
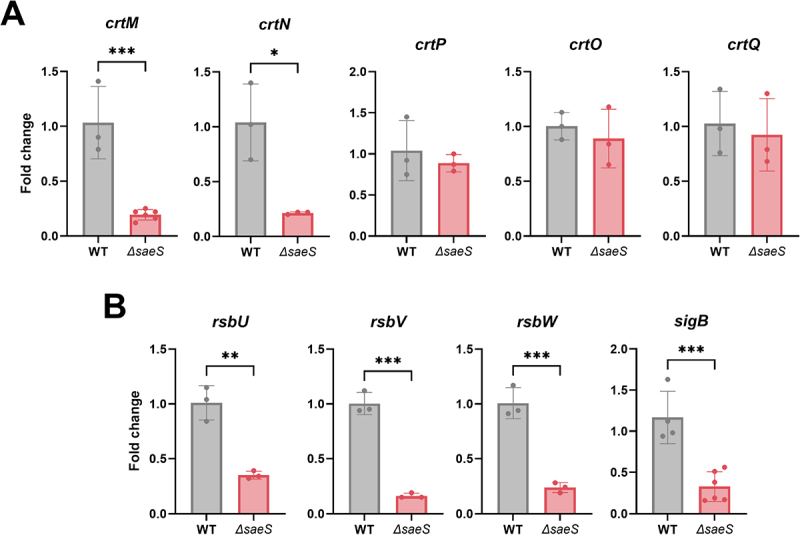
*SaeS* deletion suppresses gene expression in *rsb* and *crt* operons in *S. aureus*. Expression of genes in the *crt* (A) and *rsb* (B) operons. Significant differences between groups were assessed using Student *t*-test and are indicated by asterisks (P-values: *  < 0.05, ** < 0.01, and *** < 0.001). Data are presented as mean ± SD of biological replicates from three independent experiments.

STX biosynthesis is encoded by genes in the *crtOPQMN* operon, which contains a SigB-dependent promoter upstream of *crtO*. *sigB* expression involves the *rsbUVWsigB* system, which is necessary for *S. aureus* pigmentation [33–35]. Therefore, we evaluated the mRNA expression levels of the *crtOPQMN* and *rsbUVWsigB* systems to determine their involvement in reduced STX biosynthesis. qRT-PCR analysis of the *crtOPQMN* operon revealed that *saeS* deletion significantly downregulated *crtM* and *crtN* expression (Figure 3(A)). Among the five genes located in the *crt* operon, *crtM* and *crtN* are essential because they encode enzymes for the formation of dehydrosqualene from farnesyl diphosphate and the production of the major deep-yellow pigment 4,4’-diaponeurosporene, respectively [31]. In addition to the *crt* operon, the *rsb* operon exhibited significantly reduced gene expression in *∆saeS* (Figure 3(B)). Since SigB and RsbU are crucial for regulating STX biosynthesis and SigB activation, respectively, this finding implies that SaeRS promotes STX synthesis via the *rsb* and *crt* systems.

**Figure 4.**
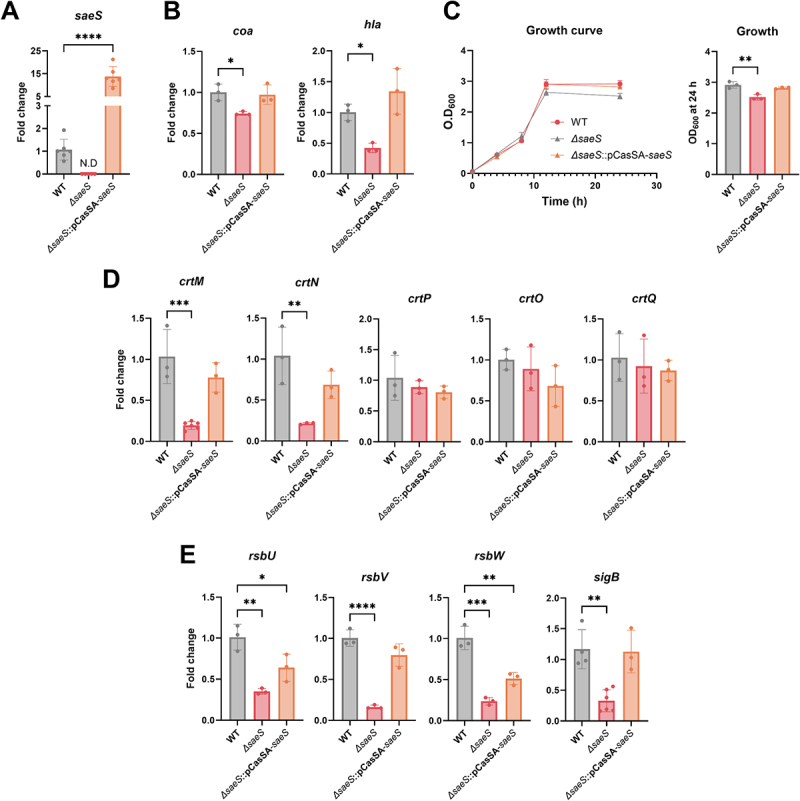
*SaeS* complementation restored virulence gene expression in *S. aureus*. (A) Restoration of *saeS* mRNA expression in the *Δsaes*-complemented strain. (B) Restoration of *coa* expression for coagulation and *hla* expression for hemolysis in the *Δsaes*-complemented strain. (C) Restoration of growth defects in the *Δsaes*-complemented strain. (D and E) Restoration of *crt* and *rsb* expression for STX synthesis in the *Δsaes*-complemented strain. One-way anova with Dunnett post hoc analysis was used to assess significant differences among the groups (P-values: *  < 0.05, ** < 0.01, *** < 0.001 and **** < 0.0001). N.D, not detected. Data are presented as mean ± SD of biological replicates from three independent experiments.

To validate the effect of SaeS on virulence gene expression, we complemented SaeS in *ΔsaeS* and assessed the expression of virulent genes. Notably, *saeS* expression was high in the complemented strain, which was controlled under the strong *rpsL* promoter using the pCasSA vector as a backbone (Figure 4(A)). *saeS* complementation restored the expression of virulence factors via increased expression of *coa*, *hla*, and the genes in the *crtMN* and *rsbUVWsigB* operons as well as the growth defect (Figure 4(B–E)). Collectively, these data are the first to show that SaeS controls STX biosynthesis by activating SigB-dependent STX production pathways.

### SaeS deletion protects mice against *S.* *aureus* bloodstream infection

**Figure 5.**
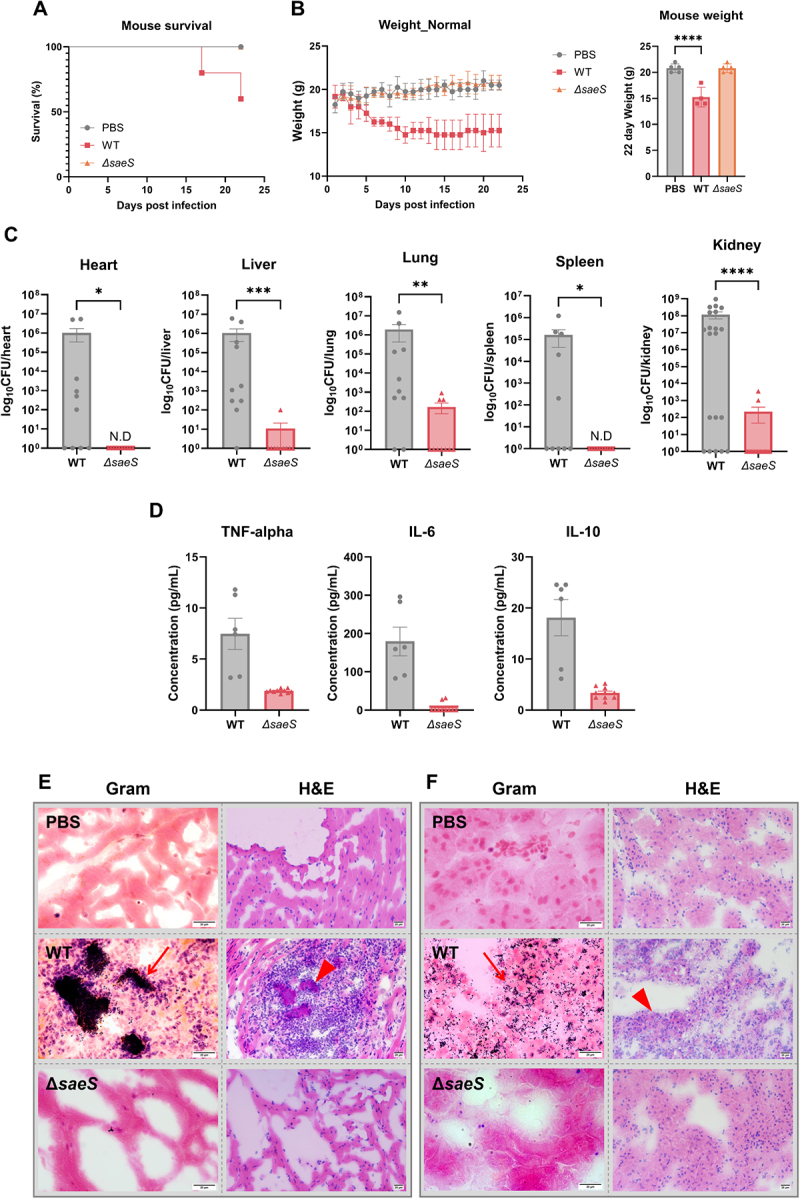
SaeS is crucial for *S. aureus* bloodstream infection in mice. (A) Survival rate in mice with *S. aureus* infection (n = 5). The mouse survival rate in the different groups was compared using the log-rank test. (B) Changes in mouse body weight from 0 to 22 days post infection with *S. aureus* WT and *ΔsaeS*. (C) Comparison of bacterial burden following bloodstream infection with *S. aureus* WT and *Δsaes* in heart, liver, lungs, spleen, and kidneys. Organs, tissues, and blood were collected and analyzed at the experimental endpoint on day 22. (D) Comparison of levels of cytokines TNF-α, IL-6, and IL-10 following infection with *S. aureus* wt and *ΔsaeS*. (E and F) Histological analysis of heart sections (E) and kidney sections (F) using Gram or hematoxylin/eosin (H&E) staining. *S. aureus* (arrow) and infiltrated immune cells (arrowhead) are indicated. Scale bar = 20 μm. Control mice were injected with phosphate-buffered saline (PBS) instead of bacteria. One-way anova with Dunnett post hoc analysis and Student *t*-test were used to assess significant differences (*p*- or *p*-values: *  < 0.05, ** < 0.01, *** < 0.001 and **** < 0.0001). N.D, not detected. Data are presented as mean ± SD of biological replicates from three independent experiments.

To assess the impact of SaeS on systemic *S. aureus* infection, a bacteremia model was established in C57BL/6 mice using PBS, *S. aureus* WT, or *∆saeS* [36,37]. The survival of mice infected with the WT strain slightly decreased at 16 days after inoculation (Figure 5(A)). Furthermore, WT-infected mice showed significantly decreased body weight compared with *∆saeS*-infected mice during the first 10 days post infection and then remained stable (Figure 5(B)). Quantification of the WT strain revealed a higher concentration in the kidneys compared with other organs (Figure 5(C)). Loss of SaeS function reduced *S. aureus* pathogenicity in bloodstream infections and exhibited an organ-specific effect on the bacterial burden, showing a greater dependency in the heart and spleen than in the lungs.

The expression of inflammatory cytokines TNF-α and IL-6 was significantly higher in WT-infected than *∆saeS*-infected mice, suggesting that SaeS-mediated a proinflammatory response to *S. aureus* infection (Figure 5(D)). IL-10 is a multifaceted cytokine generally known for its anti-inflammatory effects. It protects against the hyperactivation of inflammatory phagocytes in cases of acute systemic infection and also facilitates bacterial persistence and promotes intracellular bacterial survival during chronic localized infection [38]. The IL-10 levels in *∆saeS*-infected mice were significantly lower than in WT-infected mice, implying SaeS involvement in regulating bacterial persistence. Histological analysis of the infected lesions revealed more extensive inflammatory cell infiltration and a higher number of Gram-stained bacteria in WT infections compared with mock and *∆saeS* infections (Figure 5(E,F)). In summary, our data on *in vivo* murine bloodstream infection indicated that SaeS was a major contributor to *S. aureus* pathogenesis, inducing proinflammatory responses against the infection as well as supporting bacterial persistence.

### Zinc supplementation attenuates STX synthesis

**Figure 6.**
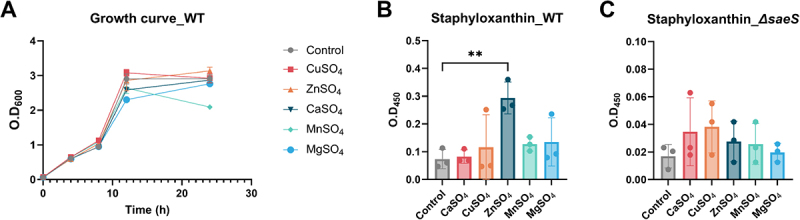
Zn supplementation alters STX synthesis in WT *S. aureus*. (A) Growth of *S. aureus* wild-type (WT). (B) STX production in *S. aureus* wt and (C) *ΔsaeS* supplemented with 100 μM of CaSO_4_, CuSO_4_, ZnSO_4_, MnSO_4_, and MgSO_4_. Metal ions were added at the time of bacterial inoculation, and cultures were incubated overnight at 37°C with shaking. Subsequent analyses were performed to assess bacterial growth and stx production. One-way anova with Dunnett post hoc analysis was used to assess significant differences among the groups (P-values: ** < 0.01). Data are presented as mean ± SD of biological replicates from three independent experiments.

Pathogens acquire essential nutrients from their host during infection [12]. In particular, dietary metal ions are highly associated with the risk of infection resulting from alterations in bacterial fitness and virulence gene expression [39]. We evaluated the effect of metal ions on *S. aureus* growth and STX production by culturing *S. aureus* Newman in RPMI 1640 medium supplemented with 100 μM of five different metal ions, namely copper (CuSO_4_), zinc (ZnSO_4_), calcium (CaSO_4_), manganese (MnSO_4_), and magnesium (MgSO_4_). None of the metal ions significantly affected the growth of WT (Figure 6(A)). In contrast, STX production increased in the presence of transition metals, specifically ZnSO_4_ (Figure 6(B)), but these effects were not observed in the *∆saeS* strain (Figure 6(C)). Zn was suggested to repress the autokinase activity of SaeS, possibly because the radius of divalent Zn (0.74 Å) is similar to Mg (0.72 Å), which acts as a cofactor for enzymatic activities [14].

**Figure 7.**
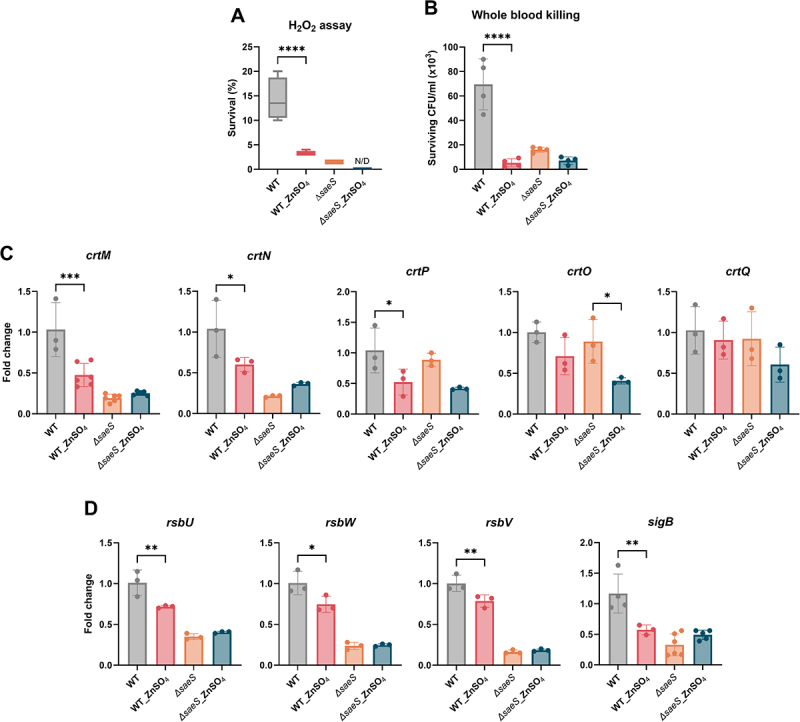
Zn supplementation attenuates*crt*and*rsb*operon expression. (A) Bacterial resistance to oxidative stress based on survival in H_2_O_2_ and (B) whole blood killing following Zn supplementation in *S. aureus* wt and *ΔsaeS*. (C and D) Expression of genes in the *crt* (C) and *rsb* (D) operons. One-way anova with Tukey post hoc analysis was used to assess significant differences among the groups (P-values: *  < 0.05, ** < 0.01, *** < 0.001, and ***** < 0.0001). N.D, not detected. Data are presented as mean ± SD of biological replicates from three independent experiments.

However, unlike the increased STX production observed following Zn supplementation, physiological analyses of bacterial survival against H_2_O_2_ and whole blood killing confirmed that Zn attenuated the bacterial resistance against oxidative stress (Figure 7(A,B)). Furthermore, Zn supplementation inhibited gene expression in the *crt* (*crtM* and *crtN*) and *rsb* operons, supporting Zn as an attenuator of gene expression associated with STX synthesis (Figure 7(C,D)). Similar to STX synthesis, the effect of Zn on the gene expression levels of the *crt* and *rsb* operons was not significantly different in the *S. aureus ∆saeS* strain, further supporting a possible association between Zn and SaeS. Notably, STX production in the Zn-supplemented WT strain was unexpectedly high despite a reduction in *crtMN* expression and antioxidative activity. There are no reports indicating an association between STX and Zn, requiring in-depth study for elucidation. Combined with our current results, we speculated that Zn inhibited the effect of SaeS and the subsequent expression of downstream genes involved in bacterial immune evasion mechanisms.

### Zn supplementation confers protection against S. aureus bloodstream infection in mice

**Figure 8.**
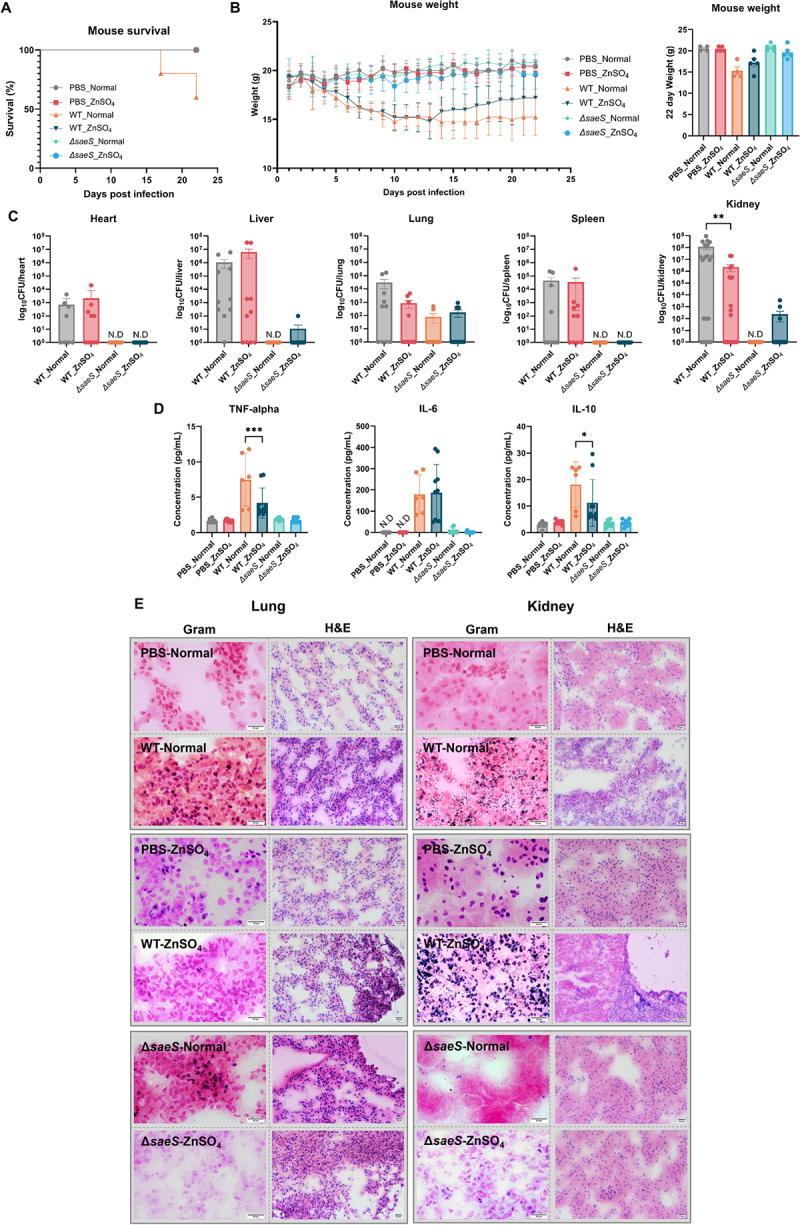
Zn supplementation supports host fitness against*S. aureus*bloodstream infection in mice. (A) Survival rate in mice with *S. aureus* infection (*n* = 5). The mouse survival rate in the different groups was compared using the log-rank test. (B) Changes in mouse body weight from 0 to 22 days post infection with *S. aureus* WTand *ΔsaeS*. (C) Comparison of bacterial burden following bloodstream infection with *S. aureus* WT and *ΔsaeS* in heart, liver, lungs, spleen, and kidneys. Organs, tissues, and blood were collected and analyzed at the experimental endpoint on day 22. (D) Comparison of levels of cytokines TNF-α, IL-6, and IL-10 following infection with *S. aureus* WT and *ΔsaeS*. (E) Histological analysis of kidney sections using Gram or hematoxylin/eosin (H&E) staining. Scale bar = 20 μm. Control mice were injected with pbs instead of bacteria. The normal, WT, and *ΔsaeS* were treated with normal water or water containing ZnSO_4_. One-way anova with Tukey post hoc analysis was used to assess significant differences among the groups (P-values: *  < 0.05, ** < 0.01, *** < 0.001, and ***** < 0.0001). N.D, not detected. Data are presented as mean ± SD of biological replicates from three independent experiments.

Because our results showed that Zn inhibited bacterial resistance against oxidative stress, we assessed the protective effect of Zn supplementation against infection with *S. aureus* WT or *∆saeS* strains (Figure 8). Zn supplementation did not induce any statistically significant toxicity during the course of the experiment without infection. However, Zn attenuated *S. aureus* pathogenesis in WT-infected mice compared with its vehicle control, increasing body weight changes (Figure 8(A,B)). Zn supplementation also resulted in lower bacterial burdens depending on the organ type, mainly in the lungs and kidneys (Figure 8(C)). Metal supplementation also reduced inflammatory cytokine levels, particularly TNF-α and IL-10 in *S. aureus* infection (Figure 8(D)). Histologically, there were no observable changes following Zn supplementation compared with the mock or *∆saeS* infections (Figure 8(E)). Taken together, we concluded that Zn supplementation conferred protection against *S. aureus* bloodstream infection *in vivo*.

## Discussion

Our investigation revealed that the histidine kinase SaeS contributes to increased adaptation and survival of *S. aureus* in the bloodstream by enhancing resistance to oxidative stress and promoting STX biosynthesis. Deletion of *saeS* significantly altered the expression of virulence genes, particularly affecting STX production via the *sigB* signaling cascade, and ultimately reduced bacterial persistence during bloodstream infection. We also found that dietary supplementation with Zn reduced bacterial antioxidative resistance by decreasing the expression of *crt* and *rsb* operon genes. Furthermore, Zn supplementation attenuated *S. aureus* infection *in vivo*, demonstrating that excess dietary supplementation with Zn inhibits the SaeRS TCS, thereby reducing bacterial infection.

The SaeRS TCS is crucial for controlling staphylococcal virulence factors. The *sae* locus was first identified in 1994 from a Tn551 mutant showing defective hemolysin, nuclease, and coagulase production [40]. Its biochemical characteristics and pathogenic mechanisms have been well documented through extensive studies [10]. Voyich et al. generated an isogenic *saeRS* deletion mutant to demonstrate that SaeRS was vital for *S. aureus* survival in a mouse model of sepsis [41]. In line with our findings, the association between hypervirulence and SaeRS was also verified in bacteremia-induced and subcutaneously infected mouse models, showing reduced mouse mortality and less severe histological lesions following infection with *S. aureus ∆saeRS* [27,42]. However, despite its clear association with virulence, the exact molecular mechanism underlying bacterial defense against the oxidative stress produced by immune cells remains unclear.

Focusing on the effects of *∆saeS* on STX production, our results suggest the feasible interaction of *saePQRS* with the *rsbUVWsigB-crtMNOPQ* pathways, which are major factors for STX biosynthesis in *S. aureus* [43]. The interaction of the Sae system with the global regulatory gene *sigB* is controversial. In some studies, the *sigB* mutant was deficient in pigmentation, coagulation, and clumping factor expression [34,44]. In contrast, microarray-based analysis of the *S. aureus sigB* regulon showed that virulence genes, including *hla*, *hlgBC*, *nuc*, and *lukFM*, were upregulated following *sigB* deletion [45]. Goerke et al. reported the absence of direct crosstalk between SaeRS and SigB, while only *sae*, but not *sigB*, showed a dominant effect on virulence expression in device-related infections [46]. Inferring the relationships between SaeRS and SigB from our current results, we verified that the Sae system affects the upstream expression of *rsbUVWsigB* and additional regulation of virulence factors, such as STX synthesis, due to reduced SigB activity.

*S. aureus* pathogenicity varies widely and is highly dependent on the presence of global regulators that control toxin production, virulence expression, and fitness factors [47]. STX is a major virulence factor that facilitates bacterial antioxidative activity by detoxifying reactive oxygen species and scavenging free radicals [48,49]. Generally, STX-producing pigmented strains tend to be more virulent, particularly in immune evasion against neutrophil killing, compared with nonpigmented strains [50]. Herbert et al. reported that STX production depended on an intact *sigB* operon, with *S. aureus* strains Newman and USA300 identified as producers of high levels of STX [47]. Being derived from the same bacterial lineage, *S. aureus* USA300 produces STX, but its virulence expression profile differs from that of *S. aureus* Newman, particularly regarding the class of SaeRS target genes [51]. Furthermore, *S. aureus* Newman has a mutation in SaeS (L18P), resulting in significantly increased kinase activity of SaeS and altered target gene expression compared with other strains [10]. Our results suggest that the balance between the SaeRS and SigB systems is important for regulating STX biosynthesis, particularly in the Newman strain. Because the Newman strain displays robust virulence properties in human and animal models [52], this finding provides mechanistic insights into the association of bacterial resistance against the immune system with STX, SigB, and SaeRS systems.

In our study, *in vitro* Zn treatment reduced bacterial resistance to oxidative stress, possibly via the *sae-rsb-crt* pathway. Recently, we also found that riboflavin (vitamin B_2_) may serve as a potential anti-virulence agent against *S. aureus*, based on in silico molecular docking predictions [53]. Similar to the suppression of STX production by Zn treatment, riboflavin also inhibited its production in a SaeS-dependent manner, supporting the current finding of an association between SaeRS and STX production. Besides its role in inhibiting bacterial virulence, Zn is a known essential micronutrient critical for regulating the host immune response to maintain host immune function [54]. In our study, Zn supplementation restored survival and body weight in mice infected with *S. aureus* while inhibiting bacterial burden and organ abscess formation, potentially enhancing tissue and organ repair. Zn is involved in multiple immunological functions, including modulation of inflammatory cytokine production [55], maturation of T-cells [56], and promotion of regulatory T-cell development [57]. In line with our results, several animal studies investigating the effects of Zn supplementation before sepsis induction demonstrated beneficial effects, including improved survival, lower bacterial burden, and lower serum concentrations of proinflammatory cytokines [58–60]. However, because most studies were based on polymicrobial sepsis, details of the mechanistic interaction between the host and bacterial systems are largely unknown. Our findings suggest that Zn supplementation may be a potential prophylactic treatment against *S. aureus* infection, simultaneously suppressing bacterial virulence and promoting immune function.

**Figure 9.**
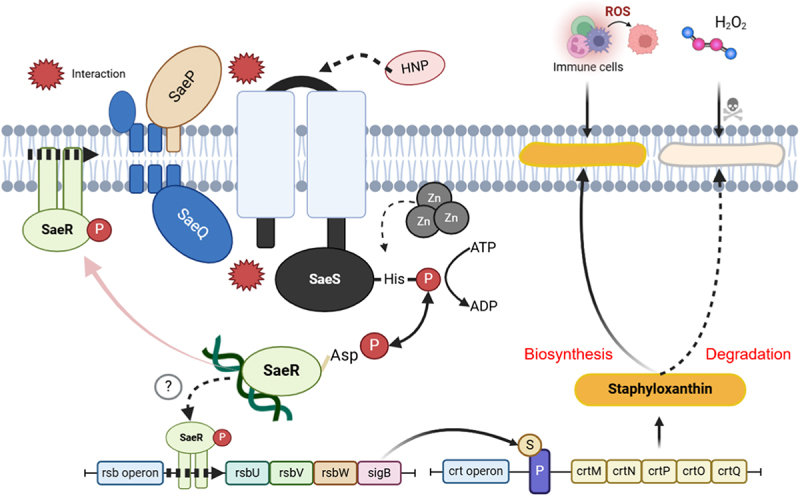
Master transcriptional regulator SaeS-mediated regulation of staphyloxanthin biosynthesis and oxidative stress resistance in *S. aureus*. schematic diagram of this study describing SaeS-mediated regulation of staphyloxanthin biosynthesis and oxidative stress resistance in *S. aureus*. The histidine kinase SaeS senses host-derived signals such as human neutrophil peptides (HNPs) and activates the SaeRS two-component system through autophosphorylation. Phosphorylated SaeS transfers the phosphate to the response regulator SaeR, which induces virulent gene expression. In parallel, SaeRS signaling is suggested to positively regulate the *rsb* operon, potentially leading to activation of the *crt* operon responsible for stx biosynthesis. stx enhances bacterial resistance to oxidative stress and immune cell-mediated killing, which may contribute to sustained bacterial survival during bloodstream infection. Deletion of *saeS* abolishes this regulation, leading to impaired survival under oxidative conditions and in bloodstream infection. Schematic diagram was created by BioRender (created in BioRender. Bae, E.).

Our study has demonstrated a regulatory relationship between the sensor histidine kinase SaeS and bacterial oxidative stress defense (Figure 9). When SaeS autophosphorylation is activated by environmental factors, such as human neutrophil peptide, SaeR binds to its recognition site, stimulating the transcription of regulons and potentially interacting with *rsbUVWsigB*. Although there have been no previous reports of SaeR binding directly to the *rsb* operon, our *in silico* analysis suggests that SaeR may potentially bind to the *rsb* promoter region, providing insight into a possible regulatory mechanism (Supplementary Figure. S6). Increased expression of the *rsb* operon, particularly *sigB*, activates the *crt* operon and STX biosynthesis, ultimately promoting the capacity of bacteria to resist oxidative bursts. During this process, Zn suppresses SaeS-mediated bacterial defense. However, detailed association of SaeS with high levels of STX synthesis, even with decreased expression of *crt* genes under conditions of Zn supplementation, requires clarification. Collectively, our results establish a novel mechanistic interconnection between SaeRS TCS and STX biosynthesis and demonstrate that SaeRS inhibition combined with Zn supplementation attenuates *S. aureus* infection.

## Supplementary Material

Final_Revision_SI_Virulence_SaeS_Clear_V2.docx

Author Checklist_Arrive_Guideline.pdf

## Data Availability

The data that support the findings of this study are openly available in Harvard Dataverse at https://doi.org/10.7910/DVN/L0YXO1.
