# Supplementary material for: Master transcriptional regulator SaeS in Staphylococcus aureus contributes to staphyloxanthin biosynthesis to promote survival during invasive infection
Source: Virulence. 2025 Oct 31;16(1):2580159. doi: 10.1080/21505594.2025.2580159 (PMC12582123; doi:10.1080/21505594.2025.2580159)

Supporting Information for

**Master transcriptional regulator SaeS in *Staphylococcus aureus* contributes to staphyloxanthin biosynthesis to promote survival during invasive infection**

Eunhwan Bae et al.

Corresponding author: Minhye Shin

Email: [mhshin@inha.ac.kr](mailto:mhshin@inha.ac.kr)

**This PDF file includes:**

Supplementary Tables S1 to S2

Supplementary Figures S1 to S6

Supplementary Table S1. Bacterial strains and plasmids used in this study

| Strain or Plasmid | Description | Reference |
| --- | --- | --- |
| Strain | | |
| *E. coli* DH5α | Cloning strain | - |
| *S. aureus* RN4220 | Laboratory strain | - |
| *S. aureus* Newman | Laboratory strain | - |
| *S. aureus* Newman-*ΔsaeS* | Newman *ΔsaeS*::*erm* | This study |
| *S. aureus* Newman-*ΔsaeS*_pCasSA-*saeS* | SaeS mutant harboring pCasSA-*saeS* | This study |
| *S. aureus* Newman_ pCasSA *saeS* | WT strain harboring pCasSA-*saeS* | This study |
|  |  |  |
| Plasmid | | |
| pCN-EF2132tet | Expression vector for EF2132 recombinase | Addgene #107191 |
| pCasSA-Cas9 | CRISPR/Cas9 system for genome editing  in *S. aureus* | Addgene #98211  Addgene #98211 |
| pCasSA-Cas9-*∆saeS* | Induced SaeS deletion mutant in Newman | This study |
| pCasSA-*saeS* | SaeS overexpression with pCasSA; Δ*cas9*::*saeS* | This study |

*S. aureus, Staphylococcus aureus*; and *E. coli, Escherichia coli*

Supplementary Table S2. Primers used in this study

| **Gene** | **Forward primer (5`→3`)** | **Reverse primer (5`→3`)** |
| --- | --- | --- |
| *gyrB*  *icaA*  *icaB*  *icaC*  *icaD*  *saeP*  *saeQ*  *saeR*  *saeS*  *crtM*  *crtN*  *crtO*  *crtP*  *crtQ*  *rsbU*  *rsbW*  *rsbV*  *sigB*  *hla*  *coa* | GGTGCTGGGCAAATACAAGT  TTTCGGGTGTCTTCACTCTATTT  GCACACTGGATGGTCATCATA  TTACGAACAACACAGCGTTTC  ATGGTCAAGCCCAGACA  GGTAGACGTATAAATCTGGACCTTT  TGTTAGCGATGACTGGTACTAAA  GTTCAACTGGTTGATGATGGTATT  TTCGAACGTACATTCAGAGTAGAA  TATGGTGTTGCTGGTACAGTAG  GTGCAGGTGTCACAGGATTAG  GACAAAGAGGGCAGAGTTGAT  GAGCAAGGCCTTTGGTATGTAG  TACTGACGGCAAATGGTATGG  TATTTCAGTGGCGGCACAA  GCATCGGCAGAGTATGTAAGT  CGAAGTTAAAGTCGGTGGAGAA  TCGCGAACGAGAAATCATACA  AGATTCTTGGAACCCGGTATATG  GCCGCTTTGTTCCATTGTTAG | TCCCACACTAAATGGTGCAA  TGGCAAGCGGTTCATACTT  CACGGTGATAATTTAATGCCAGAG  AACCCATATATGCACCTAAGAAGA  GCGATAAGTGCTGTTTCTCTTA  CACCTAACAGGTACATTCAGTTCTA  TCGTGTGGGTTCAGGTATTG  ACTTTAGGTGCAGATGACTATGTC  TTTGTTGCGCGAGTTCATTAG  GCAACGATTCACCAAGTCTTC  CATACGCCCGCCTACATTATT  TCGGTCGATTATAGCGTTGC  CCCGTCACACGTCTTTGATATG  CATCAGCATCTACAAAGGCAATG  AATGACATCTGCAACAGCAAAG  CTAACTGCAATCTTGGCATCTTC  CGAGTTCCATCTTGTCTCATAGG  CCGTTCTCTGAAGTCGTGATAC  CTGTAGCGAAGTCTGGTGAAA  TCCACAGGGCACAATTACAG |

*
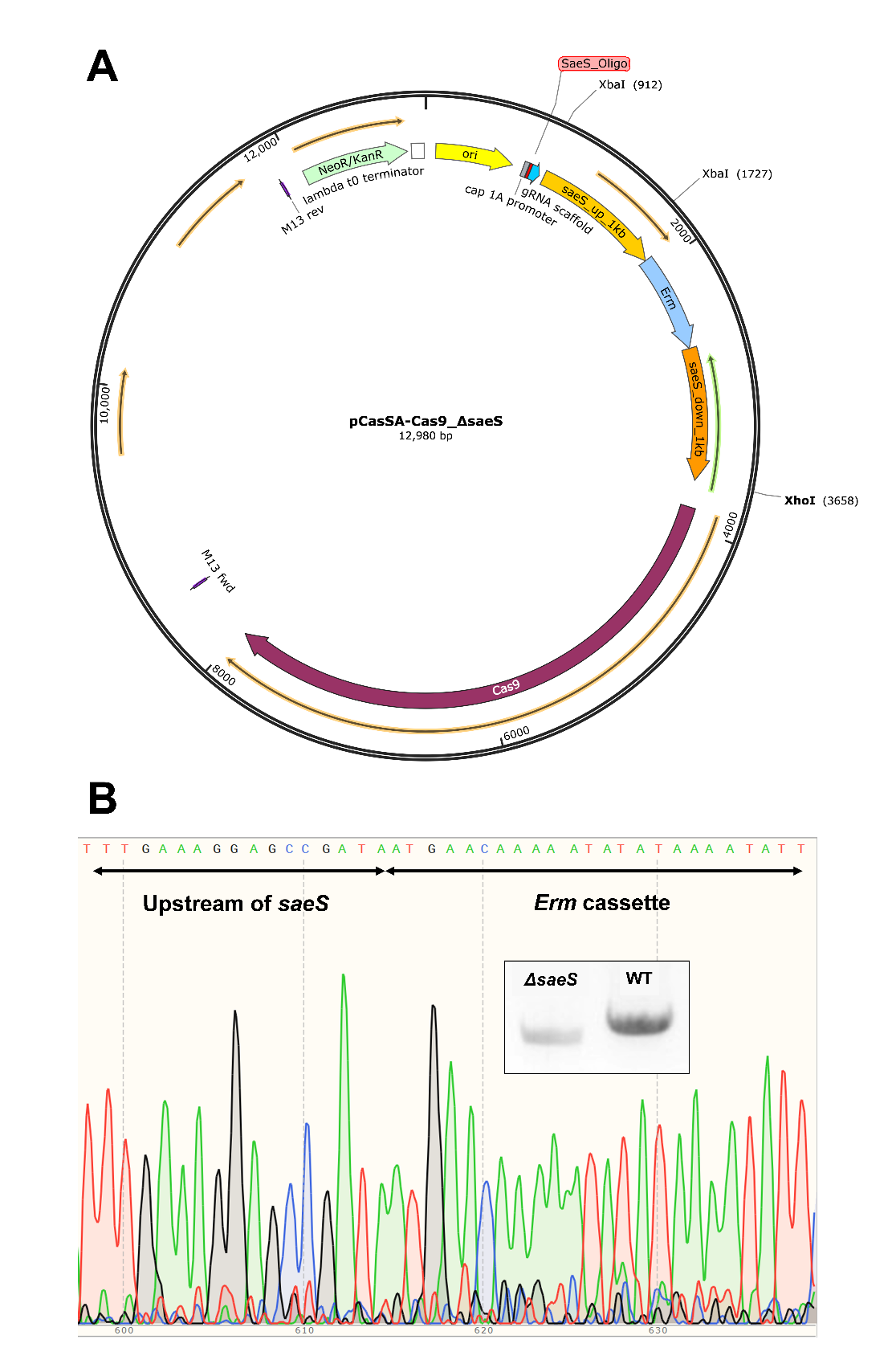
*Supplementary Fig. S1. Generation of *saeS*-null mutant (*ΔsaeS*) strain. (A) Vector map of pCasSA-*ΔsaeS*. (B) Confirmation of the *saeS* deletion by replacing the gene with erythromycin resistance gene cassette.


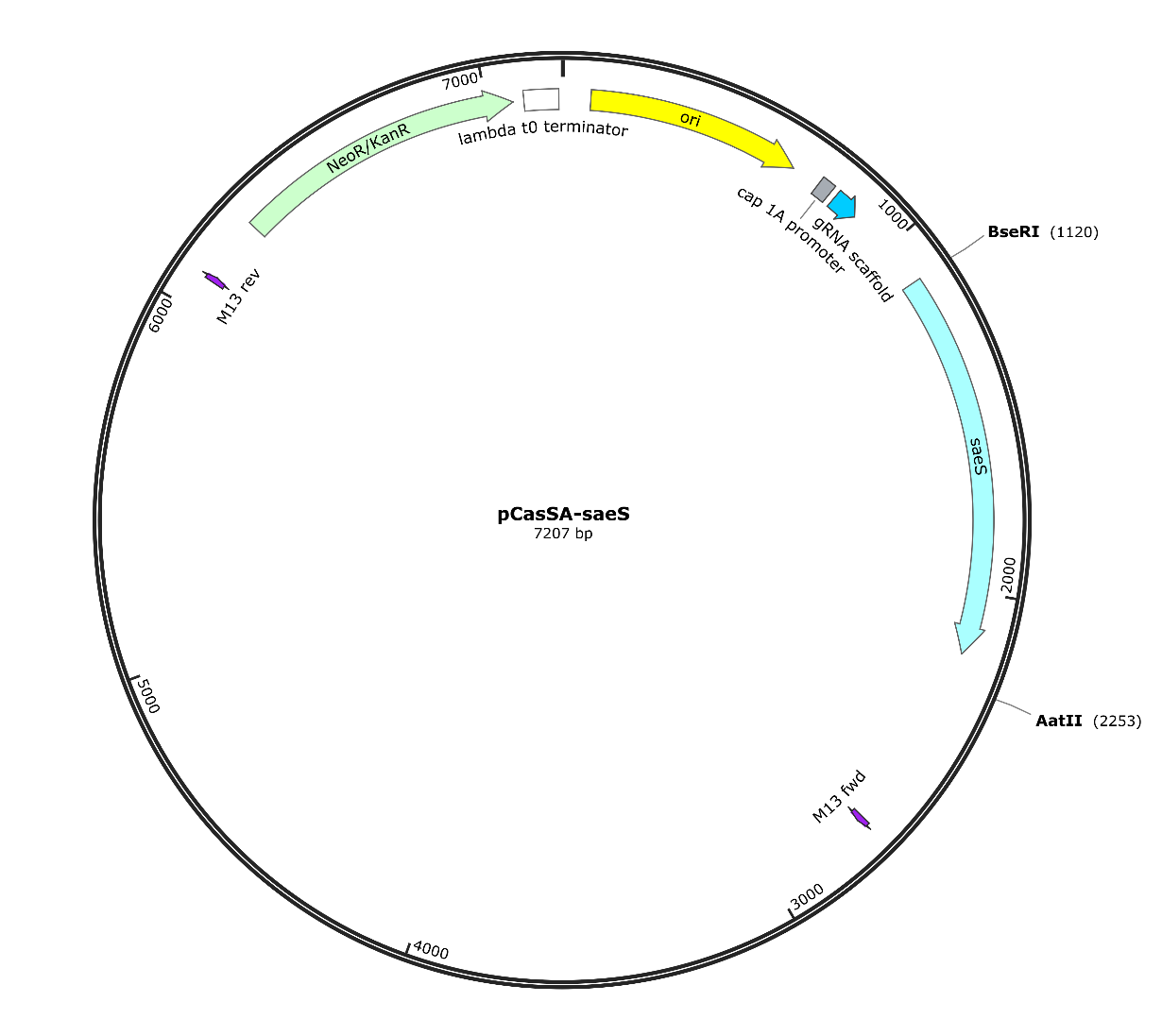
Supplementary Fig. S2. Generation of SaeS-overexpressing (pCasSA-*saeS*) strain. (A) Vector map of pCasSA-*saeS*.

**Supplementary Fig. S3.** ***saeS* mutation in *S. aureus* suppresses virulence.** Previously, we obtained two colonies exhibiting growth defects on blood agar medium, presenting as pinpoint colonies with impared STX biosynthesis, antioxidant defense, and hemolysis. Whole genome sequencing (WGS) followed by single nucleotide polymorphism (SNP) analysis revealed that both clones harbored a mutation in the saeS gene. (A) Colony morphology of wild-type (WT) and mutant strains. (B) STX production, H_2_O_2_ killing, and hemolysis in WT and mutant strains. One-way ANOVA with Dunnett post hoc analysis was used to assess significant differences among the groups (P-values: ** < 0.01, *** < 0.001, and **** < 0.0001). Data are presented as mean ± SD of biological replicates from three independent experiments. (C) List of mutated genes in the mutant strains. After WGS, the resultant 4.6-4.7 million of single-end reads from the wild-type (WT) and two mutant strains were aligned using *S. aureus* Newman as the reference genome. Comparison of the genomes of the two mutants with their parent WT revealed eight and nine single-nucleotide variants for mutant 1 and 2, respectively. (D) Predicted SaeS structure of WT and mutant strains. Major variations occurred in *saeS*, which encodes histidine kinase SaeS, a major protein in the two-component transcriptional regulator crucial for pathogenicity. Nonsense mutations were present on E319 of mutant 1 and E211 of mutant 2, with additional mutations from L202 to Y210. Mutated residues are indicated as blue and red region with side chains shown as a ball and stick model.


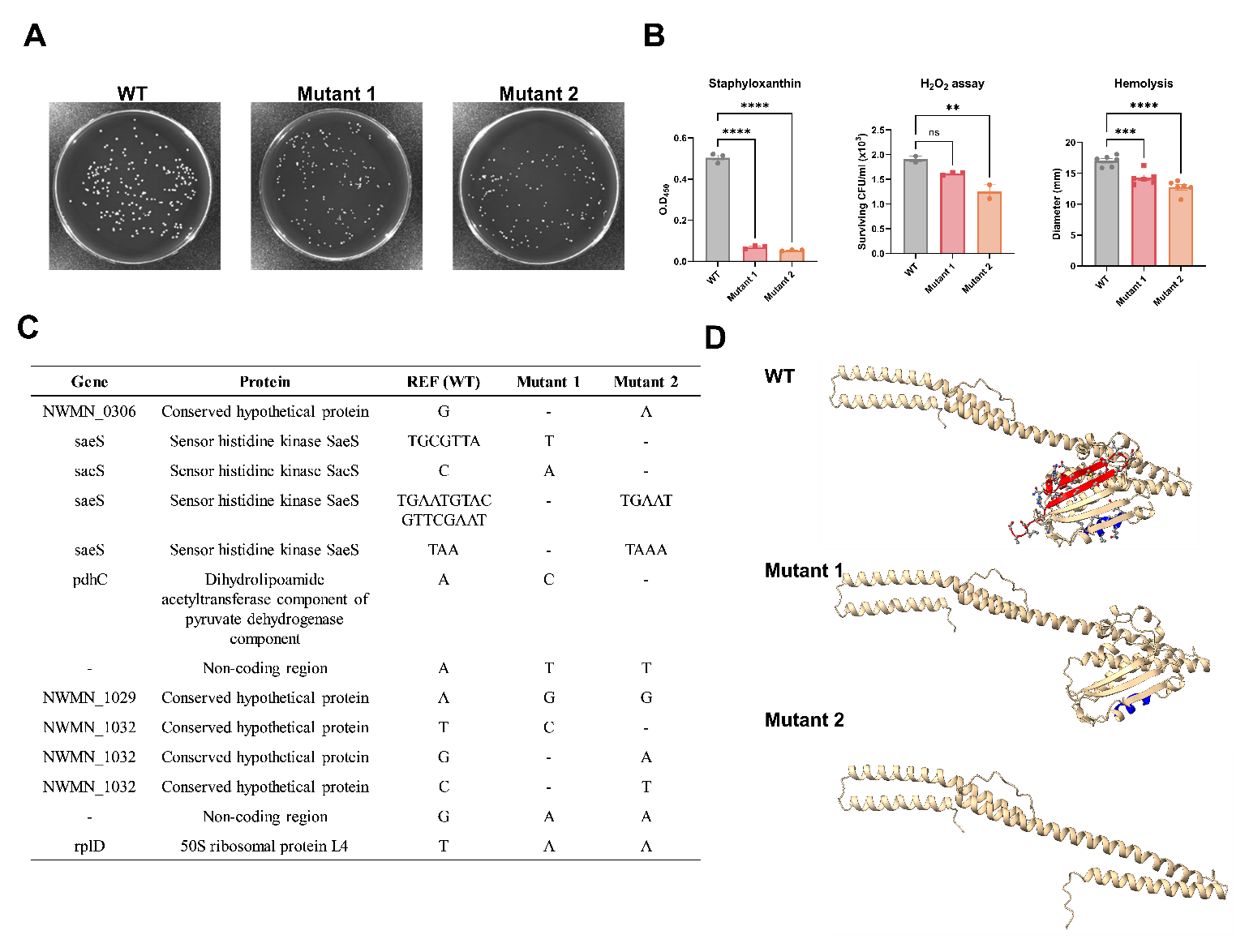


Supplementary Fig. S4. *saeS* deletion suppresses resistance to phagocytic killing in *S. aureus* Newman (WT), *ΔsaeS*, and *ΔsaeS*-complemented strains. (A) Whole blood killing to assess immune-mediated bacterial clearance. (B) Plasma killing to assess complement-mediated bacterial clearance. One-way ANOVA with Dunnett post hoc analysis was used to assess significant differences among the groups (P-value: * < 0.05). Data are presented as mean ± SD of biological replicates from three independent experiments.


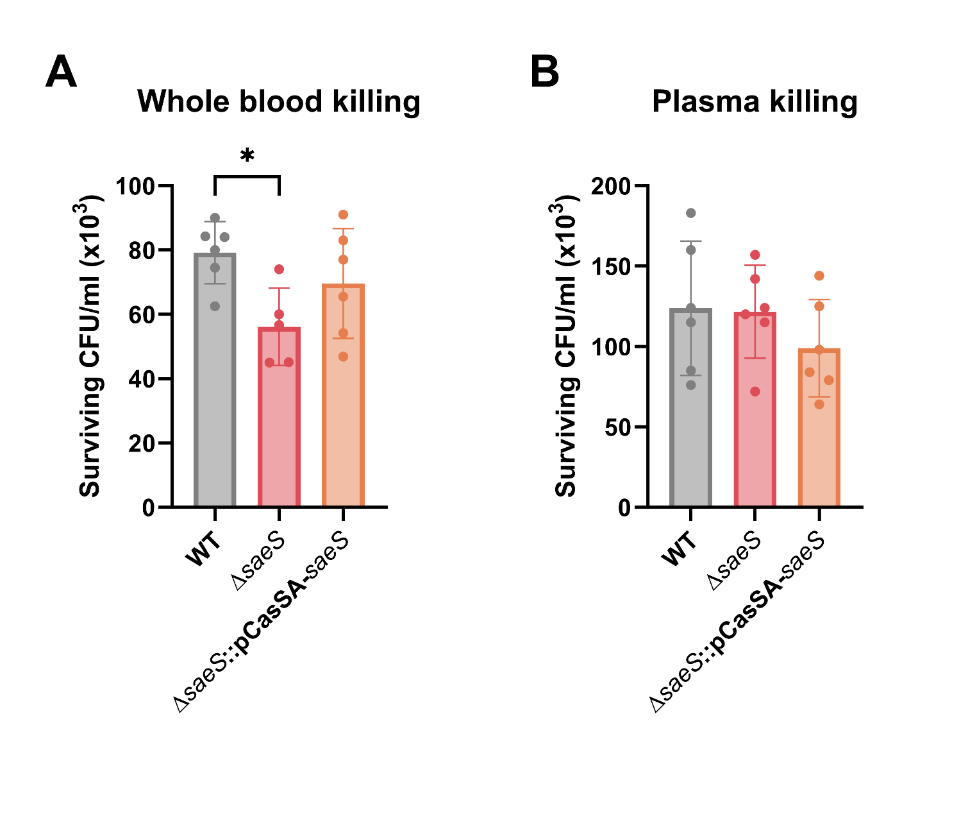


Supplementary Fig. S5. Evaluation of oxidative stress resistance in the *saeS* deletion using catalase activity. Catalase activity analysis of *S. aureus* Newman (WT), *ΔsaeS*, and the *ΔsaeS*-complemented strain. One-way ANOVA with Dunnett post hoc analysis was used to assess significant differences among the groups. Data are presented as mean ± SD of biological replicates from three independent experiments.


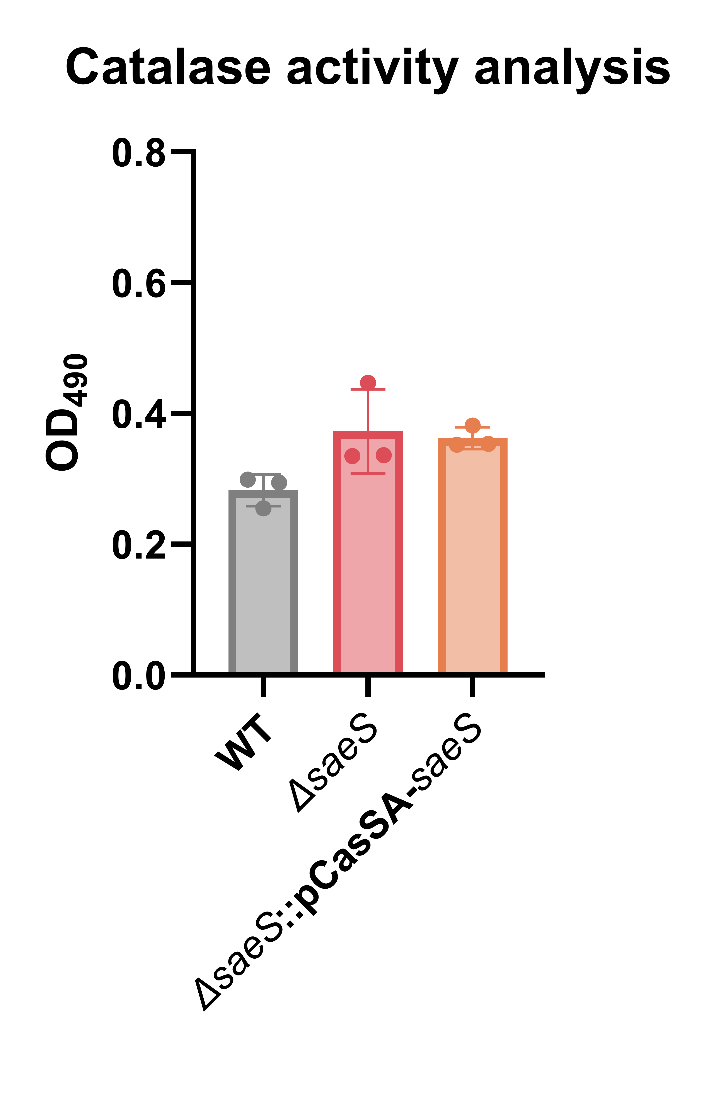


**Supplementary Fig. S6.** **Prediction of SBS (SaeR Binding Site) interaction using the HDOCK server.** A) Predicted SaeR-*saeP* binding model_1 generated by HDOCK and visualized using Discovery Studio Visualizer software. B) Sequences of SaeR-binding site (SBS) for the 12 genes, including a definite SBS region (*saeP, hla, coa, fnbB, capA, ssr/emp, hlgC, lukA*) and an indefinite SBS region (*rsb, crt, ica, walRK*). C) Summary of the Top 10 models. **Docking scores** were calculated using the ITScorePR function, where more negative values suggest higher binding potential, though not equivalent to true binding affinity. **Confidence scores** were derived empirically from docking scores, where values >0.7 indicate likely binding, 0.5–0.7 suggest possible binding, and <0.5 suggest unlikely binding. **Ligand RMSD** reflects deviation from the input or modeled structure, but is not a direct measure of docking accuracy. **Red**: SBS (SaeR binding sequence); **Blue**: SaeR (PDB ID: **4QWQ**).


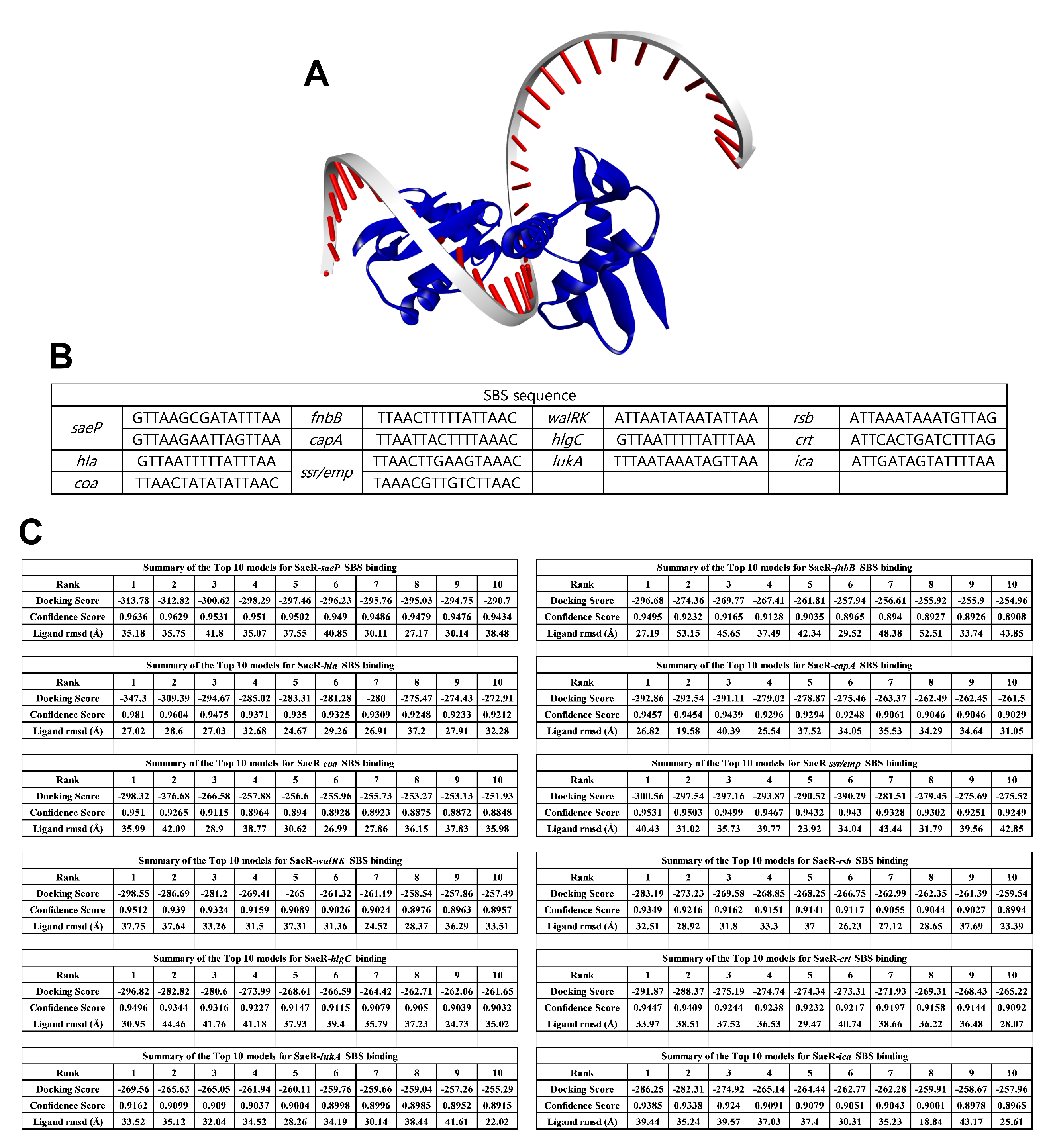

Supplement: Final_Revision_SI_Virulence_SaeS_Clear_V2.docx [file KVIR_A_2580159_SM6701.docx]
